# Clinical Audit on Identification, Evaluation and Optimisation of Anti-Cholinergic Burden in Older Adults With Cognitive Impairment Referred to East Hub Older Adult Community Mental Health Team at BSMHFT

**DOI:** 10.1192/bjo.2025.10558

**Published:** 2025-06-20

**Authors:** Sanhita Bhattacharya

**Affiliations:** Black Country Healthcare NHS Foundation Trust, Sandwell, United Kingdom

## Abstract

**Aims:** This audit aims to assess whether the anticholinergic burden is being appropriately considered in the management of patients referred with cognitive difficulties to East Hub Older Adult community mental health team.

**Methods:** A retrospective audit was conducted on the medical records of 49 patients referred from the Memory Assessment Clinic to the East Hub Older Adult CMHT over a one-year period. Data was collected using an audit tool that included patients with diagnosed dementia or cognitive decline, excluding those with substance abuse disorders. The primary focus was on whether anticholinergic burden (ACB) scores were assessed and whether medication regimens were optimized in line with NICE guidelines. The ACB score was calculated using acbcalc.com

**Results:** The audit revealed that 24% of patients with an ACB score greater than 3 had no documented evidence of an assessment of their anticholinergic burden or any medication optimization. This suggests that the East Hub Older Adult CMHT is not consistently adhering to NICE guidelines in the management of anticholinergic medications in older adults at increased risk of cognitive decline.

**Conclusion:** This audit highlights the need for more rigorous evaluation of anticholinergic drug use in our clinical practice to reduce the risk of cognitive decline in older adults. It underscores the importance of anticholinergic medications as a modifiable risk factor for dementia, emphasizing the need for healthcare providers to prioritize reducing anticholinergic burden in this population. The findings suggest that alternative medications should be considered for patients with high anticholinergic burden. These results were disseminated Trust-wide, with a plan to conduct re-audit to evaluate whether changes have been implemented in our clinical practice.

An advanced tool to calculate the ACB score using medichec.com was agreed by the clinical lead of Dementia and Frailty for use in daily clinical practice by the various older adult clinical teams in BSMHFT.

